# Factors influencing patient experience in hospital wards: a systematic review

**DOI:** 10.1186/s12912-024-02054-0

**Published:** 2024-08-01

**Authors:** Tingyu Guan, Xiao Chen, Junfei Li, Yuxia Zhang

**Affiliations:** 1 School of Nursing, Fudan University, Shanghai, China; 2 Department of Nursing, Fudan University Zhongshan Hospital, Shanghai, China

**Keywords:** Patient experience, Inpatient, Influencing factors, Systematic review

## Abstract

**Background:**

Patient experience plays an essential role in improving clinical effectiveness and patient safety. It’s important to identify factors influencing patient experience and to improve quality of healthcare.

**Objective:**

To identify factors that influence patient experience in hospital wards.

**Methods:**

We conducted a systematic review including six databases; they were PubMed, CINAHL, Embase, PsycInfo, ProQuest, and Cochrane. Studies were included if they met the inclusion criteria. The JBI checklist was used to perform quality appraisal. We used 5 domains of the ecological model to organize and synthesize our findings to comprehensively understand the multi-level factors influencing the issue.

**Result:**

A total of 138 studies were included, and 164 factors were identified. These factors were integrated into 6 domains. All domains but one (*survey-related factors*) could be mapped onto the attributes of the ecological framework: *intrapersonal, interpersonal, institutional, community, and public policy level factors*. All factors had mixed effect on patient experience. The intrapersonal level refers to individual characteristics of patients. The interpersonal level refers to interactions between patients and healthcare providers, such as the caring time spent by a nurse. The institutional level refers to organizational characteristics, rules and regulations for operations, such as hospital size and accreditation. The community level refers to relationships among organizations, institutions, and informational networks within defined boundaries, such as a hospital located in a larger population area. Public policy level refers to local, state, national, and global laws and policies, including health insurance policies. The sixth domain, survey-related factors, was added to the framework and included factors such as survey response rate and survey response time.

**Conclusion:**

The factors influencing patient experience are comprehensive, ranging from intrapersonal to public policy. Providers should adopt a holistic and integrated perspective to assess patient experience and develop context-specific interventions to improve the quality of care.

**PROSPERO registration number CRD42023401066:**

**Supplementary Information:**

The online version contains supplementary material available at 10.1186/s12912-024-02054-0.

## Introduction

Patient experience refers to “the sum of all interactions, shaped by an organization’s culture, that influence patient perceptions, across the continuum of care“ [1]. In the course of health care, patients can provide more direct and detailed information, helping providers to be more sensitive and responsive to the specific needs of individual patients, so as to provide personalized and holistic care [2]. Previous studies have indicated that better patient experience is associated with lower mortality rates, hospital-acquired infection rates, medical error rates, as well as improved health status, functional ability, and quality of life [3]. Measuring patient experience, therefore, has become a critical approach to demonstrate real changes in healthcare delivery itself and evaluate the quality of medical services which is an essential component of health service innovation [4]. The report *Crossing the Global Quality Chasm* in 2018 pointed out that the path to a high-quality future in healthcare needs to integrate elements of person-centered care into healthcare systems and continually improve the experience of patients, families, and communities [5]. Organizations and policy-makers worldwide have begun to measure, report and leverage patient experience data to implement quality improvement strategies [6].

Improving patient experience has become the common goal for global healthcare institutions, and determining the influencing factors is a necessary first step, which could lay the foundation for further intervention. Patient experience can vary in different care settings. Healthcare institutes worldwide have developed the special survey programmes targeted at different healthcare services, for example, Consumer Assessment of Healthcare Providers and Systems (CAHPS) program have released CAHPS Hospital Survey for the inpatient setting [7], and CAHPS Outpatient and Ambulatory Surgery Survey for the outpatient setting [8]. Additionally, National Accident and Emergency (A&E) Department Survey is used in the emergency setting [9]. Among different survey programs, those for the inpatient setting have attracted the most attention, since inpatients have the longest interaction time and most interaction interfaces with healthcare providers during service encounters, and their care experience will largely influence their overall rating of the hospital. Moreover, focusing on this population could contribute to achieving a more holistic and integrated perspective on the influencing factors of patient experience. However, there is no clear understanding of the extent to which various factors influence inpatients’ experience of care. Previous studies have only focused on specific populations, such as cancer patients and emergency patients [10, 11]. A systematic review addressing influencing factors for the inpatient population has been lacking.

### Theorical framework

The integration of factors influencing the patient experience was based on the ecological model proposed by McLeroy [12]. The ecological model conceptualizes health broadly, placing an individual’s behaviour in a larger context and considering multiple levels of influence external and internal to the individual, as well as interactions across levels of influence [13]. This model emphasizes five domains of influence on health outcome, ranging from micro to macro level: intrapersonal, interpersonal, institutional, community, and public policy. It is often used in various health promotion programmes because this model assumes that appropriate changes in the social environment will produce changes in individuals, and that the support of individuals is essential for implementing environmental changes.

The key reason for choosing this theory is that our aim is to provide a comprehensive depiction of influencing factors, enabling a deeper understanding of potential intervention points. Upon reviewing existing literature, we found that the influencing factors on patient experience span multiple domains, ranging from individual characteristics to policy changes. Commonly used theoretical models in patient experience research, such as Donabedian’s Structure Process-Outcome Model [14] and the Institute of Medicine’s Framework of healthcare quality [15], predominantly focus on the quality of services provided by hospitals. However, these models do not offer an intuitive and comprehensive understanding of all factors influencing patient experience.

In summary, this systematic review aims to identify influencing factors of patient experience in hospital wards within the multiple levels of McLeroy et al.‘s ecological model.

## Methods

This review was reported according to “The Preferred Reporting Items for Systematic Reviews and Meta-Analyses (PRISMA) 2020 statement“ [16]. We have registered our protocol in PROSPERO previously, the registered number is CRD42023401066.

### Search strategy

The retrieval period spans from the establishment of each of the databases to August 23, 2022 by two independent authors TG and XC. A total of six databases were searched, including PubMed, CINAHL, Embase, PsycInfo, ProQuest, and Cochrane. In addition, we supplemented the included studies by searching for citations. Search terms included Mesh terms, free-text synonyms, and controlled vocabulary for “patient experience”, “patient perception of care”, and “inpatient” to locate relevant research published. A search filter was used to limit to the English language, and there was no publication data limitation. See Additional file 1 for the detailed search strategy.

### Eligible criteria

#### Operational definition of patient experience

Although we chose articles that explicitly contained the terms “patient experience” and “patient perception of care”, the terms patient experience and patient satisfaction are often used interchangeably, with the potential to cause confusion and misunderstanding. Therefore, we carefully re-examined the articles to determine the concept of patient experience and formulated detailed criteria before reviewing the factors.

Patient experience is “feedback from patients on ‘what actually happened’ in the course of receiving care or treatment, capturing both the objective facts and their subjective views of it”. This places the focus for patient experience firmly both on what happens to patients, and how they report that experience [1, 2]. On the other hand, patient satisfaction focuses on the subjective evaluation of patients, mainly reflecting whether the care provideds meet their needs and expectations [17]. It is more an outcome variable than patient experience. Therefore, questionnaires on experience will focus on what happened and what patients felt, so the scoring method will be more objective and detailed, such as “always” and “no” rather than responses like “very satisfied” and “dissatisfied“ [2].

Based on the nature and definition of patient experience, we established the following criteria and considered a variable to be patient experience if it met all the following conditions: (1) Measurement instruments should consist of scales or questionnaires that have undergone a formal development process and have been tested for reliability or validity. (2) Likert scoring method contains frequency (never to always), agreement (disagree to agree), and degree (not at all to a very high degree), and studies with the Likert scoring method using satisfied/excellent would be excluded. (3)We focused on the studies that measured patient experience by overall scores or specific dimensions of the patient experience scale, and excluded studies that only evaluate dependent variables with specific items, global scores, and recommendation levels.

#### Inclusion and exclusion criteria

Hence, these studies would be included: (1) the population was adult hospitalized patients, (2) the outcome was patient experience, (3) the theme was examining factors associated with patient experience, (4) the study design was an observational study, (5) the article type was primary research, (6) the language was English. And studies would be excluded: (1) the research setting was in specific health facilities (pediatrics and adolescence, psychiatry, ICU, emergency, outpatient, operating room, obstetrics), (2) The study did not have any statistically significant results (*P* ≥ 0.05), (3) the full text was not available.

### Screening and data extraction

All retrieved articles were exported into Endnote X9, and duplicates were removed. Then two authors independently reviewed the studies and a consensus would be reached through discussion. The process of screening was strictly carried out according to the PRISMA flowchart [18]. The data extracted from the remaining studies included: author, date, location, sample, number of centers, design, theory framework, statistical analysis methods, outcome, outcome measurements, and factors.

### Quality evaluation

The JBI checklists for cross-sectional, cohort, and case-control studies were used to assess the methodological quality of each study [19]. Two authors (TG and JL) evaluated the study independently. If there was disagreement between the two parties, the issue would be decided by the third author (YZ). We scored yes/no/unclear/not applicated for each question, “Yes” answer scored one point, “no and unclear” scored zero point, and “not applicated” wasn’t counted. The quality score is calculated by the actual score as a percentage of the total score. Studies would be classified into the following categories: excellent (> 80%), some limitations (50–80%), and several limitations (< 50%) [20–22]. The quality appraisal wouldn’t be used as the basis for the exclusion of studies, but only for having a better understanding of the quality of the literature in the field.

### Data synthesis

We placed the collated determinants into different domain and subdomains based on the ecological model [12]. The intrapersonal level refers to individual characteristics of individuals, we divided this domain into three subdomains: patient characteristics and traits, patient health-related, and patient medical experience based on reviewed factors. The interpersonal level refers to interactions between patients and nurses, it contained two subdomains, staff’ characteristics, traits, and outcomes, staff behaviours and interactions. The institutional level refers to organizational characteristics, rules and regulations for operations. Factors in this domain were categorized into three subdomains, characteristic of institutional, organizational management model and working climate. The community level refers to relationships among organizations, institutions, and informational networks within defined boundaries. Public policy refers to local, state, national, and global laws and policies. The sixth domain of survey related factors was not part of the model. The full data synthesis process was performed by two authors(TG and XC), and the decision was made by the third author (YZ) in case of disagreement.

## Result

### Study selection

**Fig. 1:**
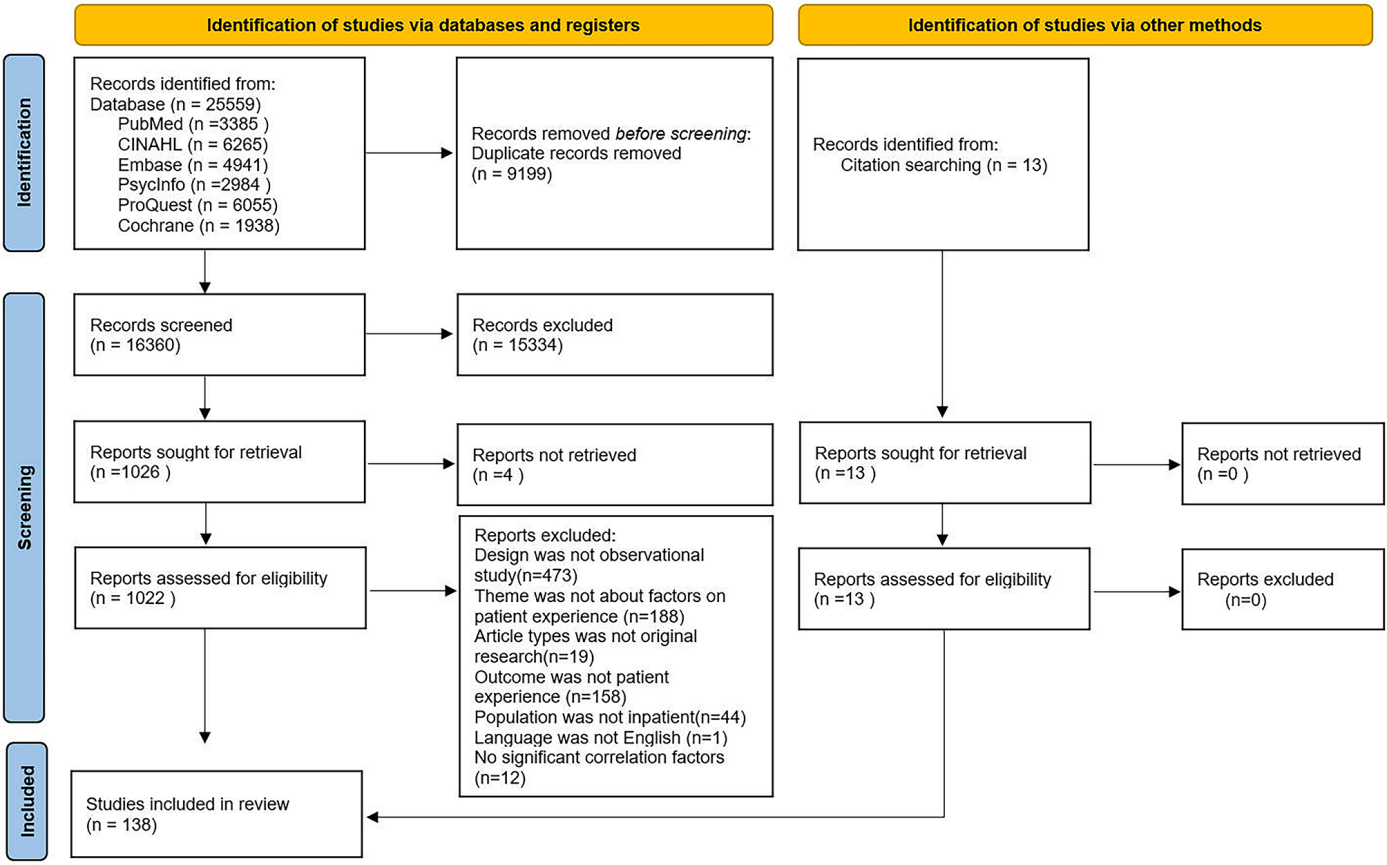
PRISMA Flowchart

A total of 25,559 studies were identified from the database, and 13 studies were identified through citation tracking. After the first round of screening 1022 papers were selected. In the second round of screening, 473 were non-observational studies(130 qualitative studies, 187 interventional studies and 156 tool development studies), 188 were not about influences on patient experience, 19 were not original research, 158 had outcomes that were not patient experience, 44 were not about inpatients, 1 was not in English, and 12 did not have any significant influences. Finally, 138 studies were included, with 133 cross-sectional, 3 longitudinal, and 2 cohort studies [23–160]. See Fig. 1**PRISMA Flowchart** for PRISMA results.

Of the total 138 studies, there were 87 studies conducted in the USA, 8 studies in the UK, 7 studies in Norway, 5 studies in China, 4 studies in the Netherlands, 3 studies in Turkey. Two studies each for countries Poland, Finland, Iran, Italy. One study each for other 13 countries, such as Denmark, German, Canada, etc. Three studies were conducted in multiple countries.

Seventy-nine studies were multicenter investigations. Hungary, Jordan, Slovenia, and Thailand had only single-center studies. There was a wide disparity in the sample size of included studies with a minimum of 50 and a maximum of 5,480,308. In addition, 38 studies did not describe the sample size.

A theoretical model was used to find possible related factors of patient experience in 12 of the 138 studies. Eight studies used *Donabedian’s Structure Process-Outcome Model*. The rest were *The Institute of Medicine (IOM)’s Framework of Patient-Centered Care, Klein and Kozlowski’s Multilevel Theory, Andersen’s Behavioral Model, Hospital Organizational Composition, Quality Health Outcomes Model, Resource Dependency Theory.* They all appeared only once.

A total of 27 measurements of patient experience were used, eight of which were patient experience of nursing (*n* = 17). The most frequently used was HCAHPS (*n* = 86). More detailed information on the characteristics was provided in Additional file 2.

### Quality assessment

A total of 133 cross-sectional studies and 3 longitudinal studies, were assessed using a cross-sectional study checklist. Additionally, 2 studies were evaluated using a cohort study checklist. The quality of 74 studies was excellent, 45 studies had some limitations and 19 studies had several limitations. See Table 1 (below).

**Table 1:** Quality Assessment of including studies

Criteria: cross-sectional and longitudinal design (*n* = 136)	No.study	
	Yes	No/UC/UA
1.Were the criteria for inclusion in the sample clearly defined?	81	55
2.Were the study subjects and the setting described in detail?	93	43
3.Was the exposure measured in a valid and reliable way?	134	2
4.Were objective, standard criteria used for measurement of the condition?	136	0
5.Were confounding factors identified?	96	40
6.Were strategies to deal with confounding factors stated?	96	40
7.Were the outcomes measured in a valid and reliable way?	135	1
8.Was appropriate statistical analysis used?	109	27
**Criteria: cohort design (***n*** = 2)**		
1. Were the two groups similar and recruited from the same population?	2	0
2. Were the exposures measured similarly to assign people to both exposed and unexposed groups?	2	0
3. Was the exposure measured in a valid and reliable way?	2	0
4. Were confounding factors identified?	2	0
5. Were strategies to deal with confounding factors stated?	2	0
6. Were the groups/participants free of the outcome at the start of the study (or at the moment of exposure)?	0	2
7. Were the outcomes measured in a valid and reliable way?	2	0
8. Was the follow up time reported and sufficient to be long enough for outcomes to occur?	2	0
9. Was follow up complete, and if not, were the reasons to loss to follow up described and explored?	1	1
10. Were strategies to address incomplete follow up utilized?	1	1
11. Was appropriate statistical analysis used?	1	1

### Factors associated with patient experience

A total of 164 factors were identified. There were 138 studies reporting at least one factor significantly related to patient experience. Age (n = 38), education (n = 27), gender (n = 43), and length of stay(n = 27) were commonly examined, but yielded mixed results – showing no influence, positive or a negative influence on patient experience. All factors were sorted into five domains by content analysis: *intrapersonal level, interpersonal level, institutional level, community level, policy level*, and we add another domain, *survey related factors*, as this was a factor related to the survey process and could not be included in the model. Tables 2–7 was presented these factors related to patient experience. In these tables, ‘increased’ and ‘decreased’ indicate whether the relationship between this factor and patient experience is positive or negative, respectively. ‘No change’ indicateds that the result is not significant(*P* ≥ 0.05).

#### Intrapersonal factors

Eighty-one factors related to intrapersonal experience were examined in 90 studies. Table 2 was presented the factors in intrapersonal level(**below**). In the *patient characteristic and traits subdomain*, the effect of most factors was mixed. Older age was positively linked to patient experience in 13 studies [39, 56, 76, 86, 89, 101, 102, 105, 106, 130, 138, 144, 157], negatively linked in 13 studies [25, 31, 37, 48, 59, 60, 68, 76, 80, 109, 143, 152, 157], and showed no change in 25 studies [24, 44, 48, 50, 54, 58, 59, 65, 68, 77, 83–86, 92, 93, 98, 102, 105, 109, 112, 113, 137, 145, 156]. The impact of factors such as higher income [49, 65, 80, 115, 126] and employed patient [49, 92, 93, 109, 116] on patient experience were inconclusive as well.

In the *patient health-related subdomain*, good health condition was the most frequent factor positively linked to patient experience in 11 studies [35, 64, 76, 86, 105, 130, 137, 143, 148, 152, 157], negatively associated in 8 studies [39, 56, 83, 84, 100, 118, 137, 138], and no significant in 7 studies [24, 49, 92, 93, 98, 145, 150].Patient experiencing complication [48, 79, 118, 134, 146] and having comorbidity or chronic disease [44, 77, 80, 92, 93, 138, 152, 159] in most studies had been negatively related to patient experience.

In the *patient medical experience subdomain*, length of stay was the most frequent factor with mixed effect on patient experience [24, 25, 42, 48–50, 58, 59, 65, 66, 68, 77, 80, 84–86, 92, 93, 98, 105, 110, 125, 129, 142, 143, 145, 153]. Both routine admission and discharge to home were positively associated with patient experience in most related studies [39, 56, 77, 105, 119, 124, 130, 139, 157]. In addition, patient isolation and number of admissions were negatively influencing factor [39, 56, 127, 149].

**Table 2:** Factors in intrapersonal levels

Factors	Significantly Increased	Significantly Decreased	No change
**Patient characteristics and traits**			
Gender(Male)	[25, 28, 31, 49, 56, 58, 61, 76, 106, 110, 130, 138, 141, 143, 150, 152, 154, 157]	[88]	[24, 44, 48, 50, 54, 58, 59, 65, 68, 77, 83–86, 92, 93, 98, 102, 105, 109, 112, 113, 137, 145, 156]
Older age	[39, 56, 76, 86, 89, 101, 102, 105, 106, 130, 138, 144, 157]	[25, 31, 37, 48, 59, 60, 68, 76, 80, 109, 143, 152, 157]	[24, 44, 50, 54, 65, 83, 85, 92, 93, 98, 110, 115, 137, 145]
Respondents with multiple races	[157]		[24, 54, 58, 77, 83, 86, 115]
Hispanic	[23, 80, 157]	[80]	
Black or African American	[23, 48, 59, 64, 70, 78, 80, 110, 150]	[76]	
White	[106]	[76]	
Other races	[103, 157]	[23, 76]	
Higher Education	[31, 49, 56, 105, 109, 116, 152]	[31, 39, 49, 56, 64, 76, 84, 103, 115, 130, 145, 153, 156, 157]	[25, 65, 73, 83, 85, 86, 93, 102, 137]
Higher Income	[80]	[80, 115, 126]	[49, 65]
Satisfied with their income	[109]		
Higher SES(Socioeconomic Status) index score		[110, 150]	
English preferring	[152, 157]	[86, 114]	
Employed patient	[109, 116]	[93]	[49, 92]
Living with family/significant	[31, 68, 93, 118]		[92]
Married status	[58, 59, 68, 116]	[93]	[49, 84, 92, 102, 109, 110, 150]
Single status		[26]	
Higher BMI	[58]		[48, 59, 68, 83, 98]
Smoking status	[49]		[59, 68, 110, 150]
Alcohol use		[150]	[44, 49]
Living in the hospital area		[105]	
From a more socioeconomically deprived area	[106, 117]		[66, 76]
Insurance type(Private)	[44]		[59, 86, 110, 150]
Have medical insurance benefits	[153]	[49]	
Medically indigent status	[78]		
Higher illness acceptance	[85]		
Higher self-esteem	[85]		
Better patients’ attitudes toward the nursing profession	[101]		
**Patient health-related**			
Good health condition	[35, 64, 76, 86, 105, 130, 137, 143, 148, 152, 157]	[39, 56, 83, 84, 100, 118, 137, 138]	[24, 49, 92, 93, 98, 145, 150]
Depression		[80]	[145]
Anxiety	[80]	[80]	
Symptom distress	[113]		
Fatigue	[80]	[80]	
Patient experienced complication	[72]	[48, 79, 118, 134, 146]	[58]
Have comorbidity or chronic disease	[80]	[44, 77, 80, 92, 93, 138, 152, 159]	[49, 58, 94, 145]
Chronic lung diseases	[48]		
More severe congestive heart failure		[48]	
Prior cardiac surgery		[48]	
Peripheral vascular disease	[48]		
Hypertension	[80]	[80]	[48]
Paralysis	[80]		
Stroke	[80]		
Syncope	[80]		
Cognitive disease	[80]	[80]	[145]
Parkinson disease	[80]	[80]	
Epilepsy	[80]	[80]	
Phychosis	[80]	[80]	
Diagnosis type as IBD(compared with rectum cancer)		[143]	
Diverticulitis		[143]	
Cancer	[80, 144]		
Respondents with more advanced stage lung cancer	[106]		
Small cell lung cancer (compared to non-small cell lung cancer)	[106]		
Surgical	[73, 80, 157]	[76, 105, 123]	[65, 77, 102, 109]
Obstetric	[76]	[153, 157]	
Longer duration of illness		[26]	
Higher pain level		[86]	[42, 96, 98, 110, 150]
Different operation type*	[77, 150]		[68, 98, 110]
**Patient medical experience**			
Have previous hospitalization experience	[31, 116]	[130]	[49, 105]
Number of admissions		[39, 56]	
Patients admitted regularly	[157]		
Routine admission	[39, 56, 77, 105, 119, 124, 130, 139, 157]		[76]
Readmission	[58]		[142]
More time admission waiting		[66]	
Two-week wait diagnosis	[106]		
Emergency department experience within 30 days		[78]	
No intensive care unit stay	[123]		
Patient interdepartmental transfers		[104]	
Receipt of radio-chemotherapy	[80, 106]		
Medication used for pain control	[96]		[69]
All-cause harm		[120]	
Number of patients reported problems		[57]	
Night spent in the corridor		[56]	
Patient isolation		[127, 149]	
Number of consults	[58]	[123]	
Patients be involvement quality management		[71]	
Perceived shared decision-making		[84]	
Active-shared participation		[113]	
Longer length of stay	[48, 50, 65, 84, 153]	[42, 58, 59, 68, 77, 80, 85, 110, 125, 129, 142, 143]	[24, 25, 49, 66, 86, 92, 93, 98, 105, 145]
Longer duration of dialysis	[44]		
Ready for discharge	[122]		
Discharge to home	[68, 83, 98, 125, 150]		[59]
Being discharged with a psychiatric diagnosis		[78]	

* The results of univariate analysis show differences, but the exact positive and negative relationship are unclear

#### Interpersonal factors

Eighteen factors related to interpersonal had been found in 23 studies. Table 3 was prestented the factors in interpersonal level**(below)**. In the *Staff’ characteristics, traits, and outcomes subdomain*, we could find staff’s age [58] and nurses’ wage [108] were negatively associated with patient experience. Conversely, nurses’ education [95] and nurses’ job satisfaction [89] were identified as positive factors. Different doctors’ specialties and nurses’ depersonalizationcan had mixed effect on patient perceptions of medical care [32, 54, 58, 155].

In the *Staff behaviors and interactions subdomain*, eight factors such as time nurses spent [25, 33] were all related positively to patient experience.Two factors including incorrect treatment [56] and counterproductive caring behaviors [116, 155] were negatively related to patient experience.

**Table 3:** Factors in interpersonal level

Factors	Significantly Increased	Significantly Decreased	No change
**Staff’ characteristics, traits, and outcomes**			
Provider is younger in age than patient		[58]	
Higher nurse wage index		[108]	
Higher nurses’ education	[95]		
Depersonalization of nurses		[155]	[84]
Nurses’ job satisfaction	[89]		
Doctors’ specialty*	[32, 54, 58]		[27]
**Staff behaviours and interactions**			
More time nurse spent with patient	[25, 33, 43, 53]		
Respond to patients quickly	[25]		
Staff communication well	[34, 35]		
Nursing-patient interaction well	[160]		
Nurses’ awareness of patients’ needs	[25]		[84]
The help provided to families and friends	[25]		
Adequate Information provided	[25, 31]		
Incorrect treatment		[56]	
Doctors take charge of patient care	[105]		
Implicit rationing of nursing care		[30]	[75]
Counterproductive caring behaviors		[116, 155]	
Continuity in nursing assignment in older adults’ acute hospitalization	[145]		

* The results of univariate analysis show differences, but the exact positive and negative relationship are unclear.

#### Institutional level

In the domain institutional factors, 49 factors were exacted from 66 studies. Table 4 was presented the factors in institutional level (below). In the *Characteristic of institutional subdomain*, larger hospital [24, 27, 43, 44, 56, 67, 74, 80, 82, 105, 108, 133, 138, 157], non-profit ownership [24, 27, 44, 67, 74, 82, 91, 107, 108, 133, 147, 157] and teaching hospital [27, 56, 67, 74, 80, 82, 91, 94, 105, 107, 108, 117, 130, 133, 137, 140, 147, 150, 157] were the most commonly occurring factors. Their impact on patient experience, along with the other ten factors, such as hospital accreditation [29, 36, 90] were mixed. Ten factors, such as physician ownership [27, 34], positively contributed to patient experience. Twelve factors like the number of patients present daily [43, 112] were all negatively related to patient experience.

*In the organizational management model subdomain*, there are eight factors in total, with six factors such as nursing staffing [24, 33, 44, 45, 80, 89, 95, 97, 108, 121, 158] having mixed effect on patient experience.

*In the working climate subdomain*, three factors such as staffs receive support from other staffs [66] were all positively related to patient experience. The other three factors have all been shown to play no role in some studies.

**Table 4:** Factors in institutional level

Factors	Significantly Increased	Significantly Decreased	No change
**Characteristic of institutional**			
Larger hospital	[80, 105, 133]	[27, 44, 56, 67, 74, 80, 82, 108, 157]	[24, 43, 138]
Community hospital	[74]		
Physician ownership	[27, 34]		
Ownership is Non-profit	[27, 44, 67, 74, 91, 107, 108, 147, 157]	[82, 133]	[24]
Institutional control is public	[27, 34, 49, 91, 157]		[49, 111]
Higher percentage of estates and hotel services contracted out		[66]	
More number of patients present daily		[43, 112]	
Population over 65(%)		[82]	
The higher proportion of other races in inpatients		[44, 53, 74, 82, 94, 117]	
Availability of emergency services		[34]	
Hospitals with electronic health record systems	[81]		[27]
Uncompensated care cost		[46]	
Patients with an activated inpatient portal account	[62]		
Provide drug allergy alerts		[80]	
More expense per daygender	[78]		
Lower noise	[41]		
RN turnover rate		[53]	
Hospital accreditation	[29, 36]	[90]	
Magnet hospital	[53, 107, 136, 158]		[34]
Teaching hospital	[56, 80, 82, 91, 94, 108, 117, 130, 140, 147]	[56, 107, 130, 133, 157]	[27, 67, 74, 105, 137, 150]
Safety-net hospitals	[51]	[51, 107]	
Most Wired hospital		[107]	
Foundation hospital	[117]		
Faith-based hospital		[107]	
Catholic affiliation hospital	[88]		
Healthcare system membership hospital	[67]	[82]	
Specialty hospitals(than general medical hospitals)	[128]		
Baldrige hospital	[107]		
Sole Provider hospital	[107]		
Free-standing facility		[44]	
Large dialysis organization facilities		[44]	
System affiliation		[108, 133]	
Website overall rating	[47, 67]	[67]	
Hospital difference*	[87]		
Department difference*	[87]		
**Organizational management model**			
Hospitalists or residents participation	[52, 55]	[27]	
Higher nursing staffing level	[24, 44, 80, 89, 95, 97, 108, 121, 158]	[33, 45]	[24, 43]
Higher physician staffing level	[27, 74, 82, 133]	[74, 82]	
Higher healthcare provider staffing level	[30, 69, 75, 83]	[24]	[94]
Nursing staffing skill mix	[108]	[97]	
Higher percentage of part-time nurses to full-time nurses	[108]		[84]
Nurse Shift length ≥ 10 h		[135]	
Hospital-level care coordination strategy	[63]		
**Working climate**			
Staffs receive support from other staff	[66]		
Nurse managers’ leadership	[30, 155]		[89]
Residency learning climate	[131]		
Staff perceived patient safety culture	[69, 99, 132]		[24]
Nurse working environment	[45]		[75]
Greater hospital cultural competency	[151]		

* The results of univariate analysis show differences, but the exact positive and negative relationship are unclear

#### Community level

There were six factors related to community exacted from 11 studies. Table 5 was prestented the factors in comunity level**(below)**. A hospital located in larger population area was the most occurring factor, showing a positive effect on patient experience in two studies [67, 80], a negative effect in 7 studies [44, 56, 74, 80, 91, 94, 133], and no effect in one study [24]. Two factors such as the residents education level of patients’ community [86] were positively influencing factors.

**Table 5:** Factors in community level

Factors	Significantly Increased	Significantly Decreased	No change	
Higher residents education level of patients’ community		[86]		
Higher percentage of receiving public assistance of patients’community	[86]			
Hospitals in areas of higher per capita income		[82]		[86]
Large swings in unemployment levels in hospital located areas	[82]			[86]
Hospital located in a larger population area (metropolitan/urban)	[67, 80]	[44, 56, 74, 80, 91, 94, 133]		[24]
More facility competitive market		[44, 82]		[133]

#### Public policy level

The policies influencing patient experience were all related to payment style. Medicaid [24, 76, 80, 94] and Medicare [27, 58, 76, 78, 80] had mixed impact on patient experience. Implementation of Maryland’s global payment model [40] was a positive factor. Table 6 is showing the factors in interpersonal level **(below)**.

**Table 6:** Factors in public policy level

Factors	Significantly Increased	Significantly Decreased	No change
Implementation of Maryland’s global payment model	[40]		
Medicaid	[80, 94]	[76, 80]	[24]
Medicare	[27, 58]	[76, 78, 80]	
Government payers(government program)		[48]	

#### Survey related

Four factors were related to the survey process. Telephone survey mode [157] was positively associated with patient experience. Longer survey response time was a negative factor in three studies [39, 77, 83]. Higher survey response rates had positive effect in 2 studies [34, 117], and no effect in one study [66]. Patients with a proxy response tended to report worse patient experiences but no change after controlling for demographic differences [38]. Table 7 is showing the factors in interpersonal level**(below)**.

**Table 7:** Factors related survey

Factors	Significantly Increased	Significantly Decreased	No change
Survey mode is telephone	[157]		
Longer survey response time		[39, 77, 83]	
Higher survey response rates	[34, 117]		[66]
Patients with a proxy response		[38]	

## Discussion

This is the first known systematic review focusing on the factors of patient experience in hospital wards that have been published, and the first systematic review to make a clear distinction between patient experience and patient satisfaction as well. Eventually, we examined a total of 164 factors and integrated them into six domains.

The intrapersonal factor refers to characteristics of the individual, such as knowledge, attitudes, behavior, self-concept, skills and encompass the developmental history of the individual [12]. Our results indicate that the influencing factors of patients’ personal traits and experiential aspects account for almost half of all factors we have reviewed, underscoring their significant role. These factors are typically antecedents to the patient experience, existing before the patient interacts with healthcare services. Patient experience is feedback from patients on ‘what actually happened’ in the course of receiving care or treatment, both the objective facts and their subjective views of it [161]. This suggests that these antecedents greatly shape each patients’ subjective perception of medical experience. In other words, patient individuality is a key reason why patient experience is hard to be controlled [162]. Therefore, understanding these antecedents can help characterize different types of patients’ preferences for medical services, recognize their preferred modes of interaction, and encourage them to co-design the healthcare delivery process, thereby optimizing patient experience [163, 164].

The impact of intrapersonal factors on patient experience is the most controversial part. Some factors exhibit opposite effects in different studies, and even show no significant effects. This highlight the complexity of individual characteristics and their varying influence on patient experiences in different contexts. In addition to methodological differences in study design, sample size, and selection of potential factors, an important reason may be the failure to consider interactions among different intrapersonal factors [165]. For example, Shulman et al.‘s study indicated that the employment status of patients had a negative impact on their overall experience. This result differs from other studies, possibly due to the older age of the patient population under consideration [93].

The interaction between healthcare provider and patients is regarded as a crucial determinant in augmenting the quality of care and patient satisfaction in any specific environment [1]. Our review identified two aspect factors, namely personal characteristics of staff and staff’s behavior and interaction. The staff’s characteristics, emotions, identity can predict healthcare professionals’ intentions and interactive behaviors, which will affect the quality of service directly [166]. A lack of effective communication between patients and physicians can in turn lead to staff burnout, frustration and other negative emotions, which will impair the healthcare provider-patient relationship and patient outcomes [167]. Therefore, to enhance the interactive quality, healthcare leaders or managers should examine how employees interact with patients in a variety of situations and how effective the interactions are. Based on the assessment, appropriate resources like audit and feedback, reminders, and educational outreach should be provided to establish effective touchpoints [168]. Given the importance of the dyadic relationship in the patient experience, it is also another extremely valuable point to explore the mechanisms of patient-provider interaction and value co-creation and to standardize the entire service process [162].

What’s unique about this review is that we also delineate influences at the institutional, community, and policy levels to help us understand the process of shaping the patient experience at a more macro level. Institutional factors can shape the nature of team members’ interaction and influence the intervention’s efficacy [169, 170]. Our review identified three domains of institutional factors, including hospital characteristics, organizational management and working climate. While hospital characteristics maybe immutable, specific mechanisms impacting patient experiences can be addressed through targeted interventions to promote the medical service quality.For example, patients perceive poorer medical staff-patient communication in hospitals with a high proportion of ethnic minority patients [53, 94], which suggests that we can improve the cultural sensitivity of healthcare providers to create a trusting, connected healthcare environment [171].

External environment can have a huge impact on the operation of an entire hospital. Communities are stratified according to dimensions of socio-economic status, which may affect the individual’s health resource and health status [172]. Our review found that community members’ education level, income level, and unemployment rate all have an effect on patient experience. Meanwhile, different external characteristics, such as market competition, social and cultural context, funding environment and policy changing can affect patient experience as well [173]. By focusing on community and policy-related factors, healthcare providers can make effective adjustments and enhance medical care quality. Therefore, hospitals should examine the influencing mechanisms of external factors in a more in-depth manner so as to respond and take strategic actions according to the factors of the surrounding community immediately.

Whether survey data were accurately representative of clinical reality depends upon the patient’s or family’s ability to recall details about the hospitalization experience after discharge. Research indicated the memory of key events in the affective and emotional cognitive realms declines over time, which can result in incomplete or inaccurate responses [174]. Our review showed longer survey response time was related to poor patient experience scores [39, 77, 83]. As recommended by CMS, it is appropriate for survey to be administered between 2 and 42 days after discharge [175]. Survey patterns can affect the patient reported outcome as well [38, 176, 177], thus we suggested valid comparisons of hospital performance require some adjustment for survey patterns and patient mix [178], and future patient experience surveys should include questions of “whether is a patient’s proxy” and “reasons for choosing a proxy”.

### Limitation

There are also limitations worth noting. First, we include large number of cross-sectional studies, which is unable to demonstrate a causal relationship between these factors and patient experience directly, only offering hypotheses for future researchers to explore. Second, to ensure the credibility and consistency of the results, we ultimately chose to include only quantitative studies with statistically significant findings and exclude qualitative studies. This approach may make the results we reviewed overlook some factors that are potentially relevant to patient experience. Third, to offer general insights into patient experience factors, our combined results integrated influences from diverse cultural contexts. However, we did not conduct specific analyses for individual cultural contexts, which can lead to some factors not applying or having opposite results in different cultural settings. Besides, the use of ecological theoretical models to guide the interpretation of results may overlook some of the interaction mechanisms between internal and external environments. Finally, the exclusion of non-English literature may result in the omission of relevant literature.

### Implication

Our research has revealed that utilizing the operational definition of patient experience we employed can effectively differentiate between these articles. Hence, we strongly recommend that future articles should clearly define patient experience and patient satisfaction to obtain more objective and realistic clinical data, and develop interventions that cater to clinical needs. In addition, this review has integrated and categorized the different domains of factors, which could help researchers gain a better and more comprehensive understanding of patient experience, and provide support in selecting the appropriate list of confounding factors for studies on patient experience. However, the mechanisms of interaction between domains still need to be explored, which is a key part of developing a precise intervention plan. We have listed the possible antecedents, but we have not been able to answer the ‘why’, i.e. why patients with higher levels of education have more negative experiences, and what are the discrepancies between patients’ mental expectations and the actual interactions that lead to good or bad experiences. Understanding these mechanisms can help us target our interventions.

## Conclusion

Patient experience has become one of the most important indicators of health service quality evaluation today, identifying influencing factors of patient experience could help healthcare providers to understand and construct targeted interventions. Our review found that patient experience in hospital wards is influenced by six domains: intrapersonal level, interpersonal level, institutional level, community level, public policy level, survey-related factors. Patient age, gender, education level, patient health condition, and teaching hospital are the most frequent factors, but the specific role of these factors on patient experience remains unclear. Future research should explore the causal mechanisms shaping patient experience in specific contexts and target the construction of interventions.

### Electronic supplementary material

Below is the link to the electronic supplementary material.

Supplementary Material 1

Supplementary Material 2

## Data Availability

The datasets supporting the conclusions of this article are included within the article and its additional files.

## Declarations

### Trial and protocol registration

PROSPERO registration number CRD42023401066.
