# Supplementary material for: Factors influencing patient experience in hospital wards: a systematic review
Source: BMC Nurs. 2024 Aug 1;23:527. doi: 10.1186/s12912-024-02054-0 (PMC11295641; doi:10.1186/s12912-024-02054-0)
Supplement: Supplementary file 1 — Supplementary Material 1 [file 12912_2024_2054_MOESM1_ESM.pdf]

Additional file 1 Detailed Search Strategy

1. PubMed

#1	((((((((((((((patient experience*[Title/Abstract]) OR (patient’s experience*[Title/Abstract])) OR (inpatient experience[Title/Abstract])) OR (patients' medical experience*[Title/Abstract])) OR (patient reported experience[Title/Abstract])) OR (customer experience*[Title/Abstract])) OR (care experience*[Title/Abstract])) OR (hospital experience*[Title/Abstract])) OR (Patients' perceptions of care[Title/Abstract])) OR (HCAHPS[Title/Abstract]))) OR (Hospital Consumer Assessment of Healthcare Providers and Systems[Title/Abstract])) OR (national inpatient survey[Title/Abstract])))	3385
#2	(((((((((((((((inpatients[MeSH Terms]) OR (inpatient*[Title/Abstract])) OR (hospitalization[Title/Abstract])) OR (hospitalisation[Title/Abstract])) OR (hospitalized[Title/Abstract])) OR (hospitalised[Title/Abstract])) OR (Patient Admission[MeSH Terms])) OR (Patient Admit*[Title/Abstract])) OR (Patient Admission*[Title/Abstract])) OR (patient discharge[MeSH Terms])) OR (patient discharge*[Title/Abstract])) OR (hospital discharge*[Title/Abstract])) OR (ward[Title/Abstract])) OR ("day patient"[Title/Abstract])) OR (Day Care, Medical[MeSH Terms])) OR (medical unit[Title/Abstract])) OR ("hospital stay"[Title/Abstract])	578037
#3	((((emergen*[Title]) OR (outpatient[Title])) OR ("nurse experience"[Title])) OR ("doctor experience"[Title])) OR ("physician experience"[Title])	167663
#4	#1 AND #2	3653
#5	#4 NOT #3	3515
#6	#5 Filters: English	3385

Search until August 23, 2022

2. Ovid - EMBASE: Excerpta Medica

#1	exp hospital patient/	218770
#2	exp hospital admission/	247875
#3	exp hospital discharge/	162405
#4	exp day care/	12984
#5	"inpatient*".ab,kw,ti.	220968
#6	"hospitali*ation".ab,kw,ti.	291707
#7	"hospitali*ed".ab,kw,ti.	217169
#8	"patient amission*".ab,kw,ti.	3619
#9	"Patient Admit*".ab,kw,ti.	4808
#10	"hospital discharge*".ab,kw,ti.	53799
#11	"day patient".ab,kw,ti.	1597
#12	ward.ab,kw,ti.	70634
#13	"hospital stay".ab,kw,ti.	152692
#14	(patient adj2 discharge*).ab,kw,ti.	33668
#15	(medical adj2 unit).ab,kw,ti.	5536

#16	1 or 2 or 3 or 4 or 5 or 6 or 7 or 8 or 9 or 10 or 11 or 12 or 13 or 14 or 15	1182364
#17	Hospital Consumer Assessment of Healthcare Providers 'and' Systems.ab,kw,ti.	568
#18	hcahps.ab,kw,ti.	738
#19	national inpatient survey.ab,kw,ti.	80
#20	(patient* adj2 perceptions of care).ab,kw,ti.	195
#21	((patient* or inpatient* or care or customer or medical or hospital) adj2 experience).ab,kw,ti.	78617
#22	17 or 18 or 19 or 20 or 21	79165
#23	"emergen*".ti.	174419
#24	outpatient.ti.	39433
#25	((nurse* or doctor* or physician*) adj1 experience).ti.	778
#26	23 or 24 or 25	213877
#27	16 and 22	11406
#28	27 not 26	10929
#29	limit 28 to english language	10568
#30	limit 29 to (article or article in press or "preprint (unpublished, non-peer reviewed)" or short survey)	4941

Search until August 23, 2022

3. EBSCO - CINAHL Complete

#1	(AB (inpatient* or hospitali*ation or hospitali*ed) OR AB (Patient Admission* or Patient Admit* or hospital discharge* or "day patient" or ward or "hospital stay") OR AB (patient n2 discharge* or medical n2 unit) OR TI (inpatient* or hospitali*ation or hospitali*ed) OR TI (Patient Admission* or Patient Admit* or hospital discharge* or "day patient" or ward or "hospital stay") OR TI (patient n2 discharge* or medical n2 unit)) or MH inpatients or MH patient admission or MH day care or MH patient Discharge	282715
#2	(AB (Hospital Consumer Assessment of Healthcare Providers and Systems or hcahps) OR AB national inpatient survey OR AB Patient* W2 perceptions of care) OR AB ((patient* or inpatient* or care or customer or medical or hospital) N2 experience) OR TI (Hospital Consumer Assessment of Healthcare Providers and Systems or hcahps) OR TI national inpatient survey OR TI Patient* W2 perceptions of care) OR TI ((patient* or inpatient* or care or customer or medical or hospital) N2 experience))	52127
#3	#1 AND #2	7625
#4	emergen* or outpatient or (nurse* or doctor* or physician*) n1 experience	95111
#5	#4 NOT #3	3515
#6	#3 NOT #4 by English	6265

Search until August 23, 2022

4. Cochrane Library

#1	(Hospital Consumer Assessment of Healthcare Providers Systems or hcahps):ti,ab,kw OR (national inpatient survey):ti,ab,kw OR (Patient* NEAR/2	10350

	perceptions of care):ti,ab,kw OR ((Patient* or inpatient* or care or customer or medical or hospital) NEAR/2 experience):ti,ab,kw	
#2	(inpatient* or hospitali*ation or hospitali*ed or Patient Admission* or Patient Admit* or hospital discharge* or "day patient" or ward or "hospital stay"):ti,ab,kw OR (patient NEAR/2 discharge*):ti,ab,kw OR (medical NEAR/2 unit):ti,ab,kw	140515
#3	(MeSH descriptor: [Patient Admission] explode all trees) OR MeSH descriptor: [inpatients] explode all trees OR (MeSH descriptor: [day care] explode all trees) OR (MeSH descriptor: [patient Discharge] explode all trees)	3632
#4	emergen* or outpatient or (nurse* or doctor* or physician*) near/1 experience	19107
#5	#2 OR #3	140635
#6	(#1 AND #5) NOT #4 Limiters：In trials、Clinical Answers and Special collections	1938

Search until August 23, 2022

5. PsycInfo

#1	(AB (inpatient* or hospitali*ation or hospitali*ed) OR AB (Patient Admission* or Patient Admit* or hospital discharge* or "day patient" or ward or "hospital stay") OR AB (patient n2 discharge* or medical n2 unit) OR TI (inpatient* or hospitali*ation or hospitali*ed) OR TI (Patient Admission* or Patient Admit* or hospital discharge* or "day patient" or ward or "hospital stay") OR TI (patient n2 discharge* or medical n2 unit))	117215
#2	(AB (Hospital Consumer Assessment of Healthcare Providers and Systems or hcahps) OR AB national inpatient survey OR AB Patient* W2 perceptions of care) OR AB ((patient* or inpatient* or care or customer or medical or hospital) N2 experience) OR TI (Hospital Consumer Assessment of Healthcare Providers and Systems or hcahps) OR TI national inpatient survey OR TI Patient* W2 perceptions of care) OR TI ((patient* or inpatient* or care or customer or medical or hospital) N2 experience))	27612
#3	#1 AND #2	3295
#4	TI(emergen* or outpatient or (nurse* or doctor* or physician*) n1 experience)	28595
#5	#3 NOT #4	3169
#6	#3 NOT #4 Limiters - Language: English	2984

Search until August 23, 2022

6. ProQuest Health & Medical Complete

#1	(((mesh(inpatients) OR mesh(Patient admission) OR mesh(Day care) OR mesh(Patient discharge)) OR mesh(day care)) OR (ab(inpatient* OR hospitali*ation OR hospitali*ed OR Patient Admission* OR Patient Admit* OR hospital discharge* OR "day patient" OR ward OR "hospital stay") OR ab(patient NEAR/2 discharge*) OR ab(medical NEAR/2 unit) OR ti(inpatient* OR hospitali*ation OR hospitali*ed OR Patient Admission* OR Patient Admit* OR hospital discharge* OR "day patient" OR "in hospital" OR "hospital stay") OR ti(patient NEAR/2 discharge*) OR ti(medical NEAR/2 unit))	346462
#2	(ab(Hospital Consumer Assessment of Healthcare Providers ‘and’ Systems OR	44571

	hcahps) OR ab(national inpatient survey) OR ab(Patient* Pre/2 perceptions of care) OR ab((Patient* OR inpatient* OR care OR customer OR medical OR hospital) NEAR/2 experience) OR ti(Hospital Consumer Assessment of Healthcare Providers ‘and’ Systems OR hcahps) OR abti(national inpatient survey) OR abti(Patient* Pre/2 perceptions of care) OR abti((Patient* OR inpatient* OR care OR customer OR medical OR hospital) NEAR/2 experience))	
#3	(ti(emergen*) OR ti(outpatient) OR ti(nurse* n/1 experience) OR ti(doctor n/1 experience)) OR ti(physician* n/1 experience)	88718
#4	(S1 AND S2) NOT S3	6801
#5	Limitations: Publication type: English, academic journals, dissertations, reports	6065

Search until August 23, 2022
